# Significant Association of* HLA-B* Alleles and Genotypes in Thai Children with Autism Spectrum Disorders: A Case-Control Study

**DOI:** 10.1155/2015/724935

**Published:** 2015-12-24

**Authors:** Apichaya Puangpetch, Pongwut Suwannarat, Montri Chamnanphol, Napatrupron Koomdee, Nattawat Ngamsamut, Penkhae Limsila, Chonlaphat Sukasem

**Affiliations:** ^1^Division of Pharmacogenomics and Personalized Medicine, Department of Pathology, Faculty of Medicine Ramathibodi Hospital, Mahidol University, Bangkok 10400, Thailand; ^2^Laboratory for Pharmacogenomics, Somdech Phra Debaratana Medical Center (SDMC), Ramathibodi Hospital, Bangkok 10400, Thailand; ^3^Yuwaprasart Waithayopathum Child and Adolescent Psychiatric Hospital, Department of Mental Health Services, Ministry of Public Health, Samut Prakarn 10270, Thailand

## Abstract

Autism is a severe neurodevelopmental disorder. Many susceptible causative genes have been identified. Most of the previous reports showed the relationship between the Human Leukocyte Antigen (HLA) gene and etiology of autism. In order to identify* HLA-B* alleles associated with autism in Thai population, we compared the frequency of* HLA-B* allele in 364 autistic subjects with 952 normal subjects by using a two-stage sequence-specific oligonucleotide probe system (PCR-SSOP) method based on flow-cytometry technology.* HLA-B* ^⁎^ *13:02* (*P* = 0.019, OR = 2.229),* HLA-B* ^⁎^ *38:02* (*P* = 0.049, OR = 1.628),* HLA-B* ^⁎^ *44:03* (*P* = 0.016, OR = 1.645), and* HLA-B* ^⁎^ *56:01* (*P* = 1.78 × 10^−4^, OR = 4.927) alleles were significantly increased in autistic subjects compared with normal subjects. Moreover, we found that the* HLA-B* ^⁎^ *18:02* (*P* = 0.016, OR = 0.375) and* HLA-B* ^⁎^ *46:12* (*P* = 0.008, OR = 0.147) alleles were negatively associated with autism when compared to normal controls. Both alleles might have a protective role in disease development. In addition, four* HLA-B* genotypes of autistic patients had statistically significant relationship with control groups, consisting of* HLA-B* ^⁎^ *3905*/^⁎^ *5801* (*P* = 0.032, OR = 24.697),* HLA-B* ^⁎^ *2704*/^⁎^ *5801* (*P* = 0.022, OR = 6.872),* HLA-B* ^⁎^ *3501*/^⁎^ *4403* (*P* = 0.021, OR = 30.269), and* HLA-B* ^⁎^ *1801*/^⁎^ *4402* (P = 0.017, OR = 13.757). This is the first report on* HLA-B* associated with Thai autism and may serve as a marker for genetic susceptibility to autism in Thai population.

## 1. Introduction

Autism spectrum disorders (ASD) are neurodevelopmental syndromes characterized by early childhood onset and associated with brain abnormalities [1]. Children with autism have a pattern of behavior deficits in three major domains, namely, social interaction difficulties, verbal and nonverbal communication, and the presence of restricted, repetitive, or stereotypic behaviors or interests. The ASD was classified according to American Psychiatric Association (DSM-IV-TR) 2000 into 5 classes consisting of autistic disorder, Asperger's syndrome, Rett syndrome, childhood disintegrative disorder (CDD), and pervasive developmental disorders not otherwise specified (PDD-NOS). Clinical features usually have developmental markers of ASD emerging during the first 3 years of life. Prevalence estimates worldwide range from 0.07% to 1.8% [2] and 0.1% in Thailand [3] with a biased male-to-female ratio of 4.2 to 1 [2]. ASD has been incessantly increased so that the understanding of aetiopathogenesis of autism may be important for clinical implementation.

However, the etiology of ASD is still unclear. Genetic, environmental, and immune mechanisms in the nervous system impairments have been proposed [4, 5]. Several immune abnormalities in autistic subjects including the involvement of autoimmune disease and infection have been suggested [6–8]. Moreover, the dysregulation of the immune response such as partial T cell activation [9] and decreased peripheral lymphocyte numbers [10] as well as cytokine production [11] have been studied. The previous report demonstrated that Human Leukocyte Antigen (HLA) alleles are involved in ASD, especially HLA class I molecules playing a vital role in brain development [12–14].* HLA* genes are the name for the Major Histocompatibility Complex (MHC) in humans localized on the short arm of chromosome 6 (6p21; about 4 × 10^6^ bp).* HLA* genes are a high level of polymorphisms composed of class I, II, and III molecules. They are involved in many biological functions such as inflammation, immune response, ligands for immune cell receptors, and complement.

Previous studies demonstrated that* HLA-A* ^*∗*^ *1, A* ^*∗*^ *02, B* ^*∗*^ *07, B* ^*∗*^ *44, B* ^*∗*^ *51, DRβ*1^*∗*^ *04 (DR4), DR11, DR13, DR14, DRB*1^*∗*^ *03, DQB*1^*∗*^ *0202, DQB*1^*∗*^ *0302, DQB*1^*∗*^ *0501,* and* C4B* are associated with ASD [15]. Moreover, in 2002, Torres et al. found that* HLA-DR4* was susceptible to autism. In contrast,* HLA-DR13* has a protective role toward disease development [16]. In addition, Daniels et al. in 1995 reported that the allelic products of certain genes of the MHC were associated with autism including the null allele of the* C4B* gene (located in class III region of the MHC), the extended haplotype* HLA-DRβ*1^*∗*^ *04* (*DR4*) and* HLA-DR13* in class II, and* A2* and* HLA-B* ^*∗*^ *44* allele in class I region [17]. The evidence suggests possible associations between* HLA* alleles and ASD have wide coverage of different ethnicities. Most reports have been noted in Western countries but among Asian populations there is less information. The main aim of this study is to find out the link between* HLA-B* and ASD in Thai autistic children compared with normal subjects.

## 2. Materials and Methods

### 2.1. Subjects

A study was conducted in 1316 Thai individual subjects; 952 subjects were classified as normal control and 364 subjects were diagnosed with autistic spectrum disorder (ASD) according to the 4th edition of the diagnostic and statistical manual of mental disorders criteria (DSM-IV). All autistic subjects were recruited from Yuwaprasart Waithayopathum Child and Adolescent Psychiatric Hospital, Samut Prakan, Thailand. This study received approval from the Ethical Committee of Ramathibodi Hospital. All patients wrote informed consent document before enrolment in the project.

### 2.2. Genomic DNA Extraction

Blood samples were taken and collected to EDTA tubes. DNA was isolated by using the MagNA Pure automated extraction system (Roche diagnostics, USA) which is based on magnetic-bead technology with a lysis buffer and proteinase K. Nucleic acids are bound to the surface of the magnetic glass particles. Cellular debris was removed by several washing steps and the purified nucleic acids are eluted. From the 1 mL input volume of EDTA-whole blood, 200 *μ*L output volume of extracted genomic DNA product is obtained.

The quality of genomic DNA was assessed by using NanoDrop ND-1000 for measuring the genomic DNA as well as purity with dynamic ranges around 220 to 750 nm. Wavelength at 260 nm is suitable for measuring the genomic DNA and at 280 nm was used to evaluate contaminated protein in the sample. The recommended purified genomic DNA template for this study is 20 ng and the optical density (OD) ratio at 260/280 nm is greater than 1.7. All DNA was aliquoted and stored at −20°C until tested.

### 2.3. *HLA-B* Typing

*HLA-B* genotyping was carried out based on Polymerase Chain Reaction-sequence-specific oligonucleotides (PCR-SSO) by using the Luminex Multiplex Technology, a flow-cytometry method (Luminex IS 100, USA). Briefly, the PCR product was hybridized against a panel of oligonucleotide probes coated on polystyrene microspheres that have sequences complementary to stretches of polymorphism within the target* HLA-B* alleles. The amplicon-probe complex is visualized using a colorimetric reaction and fluorescence detection technology. Data analysis for the* HLA-B* assays was performed with software package HLAfusion 2.0.

### 2.4. Statistical Analysis

The* HLA-B* allele frequency of the samples was calculated by direct counting and calculated by dividing the total number of occurrences of that allele by the total number of alleles at that locus in the population. The significance of difference in frequencies of* HLA-B* alleles between autistic subject and control was compared by Chi-square (*χ* ^2^) test or Fisher's exact test if the number in any cell of the 2 × 2 contingency tables was less than five. Odds ratios (ORs) and 95% confidence intervals (CIs) were calculated to determine levels of significances. For all tests, a probability (*P*) of less than 0.05 was significant.

## 3. Results

### 3.1. The Characteristics of Autistic Patients and Normal Subjects

The clinical data of 1316 subjects was shown in Table 1. A mean age of 364 autistic patients was 9.17 ± 4.99 years and was divided into 314 male and 50 female (ratio 6.28 : 1), whereas the mean age of 952 normal subjects was 45.36 ± 26.15 and was classified into 677 male and 285 female (ratio 2.34 : 1).

### 3.2. *HLA-B* Allele Frequency in Autistic Spectrum Disorders Patients Compared with Normal Subjects

The* HLA-B* frequencies of 364 autistic patients and 952 normal controls were showed in Table 2. One hundred and thirty-one different* HLA-B* alleles were identified in this study. In* HLA-B* ^*∗*^ *13:02* (*P* = 0.019, OR = 2.229),* HLA-B* ^*∗*^ *38:02* (*P* = 0.049, OR = 1.628),* HLA-B* ^*∗*^ *44:03* (*P* = 0.016, OR = 1.645), and* HLA-B* ^*∗*^ *56:01* (*P* = 1.78 × 10^−4^ OR = 4.927) alleles were found to be significantly associated with autism. Two of the* HLA-B* alleles were negatively linked to autism:* HLA-B* ^*∗*^ *18:02* (*P* = 0.016, OR = 0.375) and* HLA-B* ^*∗*^ *46:12* (*P* = 0.008, OR = 0.147).

### 3.3. *HLA-B* Genotype Frequencies in Autistic Children and Normal Subjects

There were 442 different* HLA-B* genotypes in the 1316 subjects. Four* HLA-B* genotypes of autistic patients had statistically significant relationship with control groups (Table 3) which are composed of* HLA-B* ^*∗*^ *1801*/^*∗*^ *4402* (*P* = 0.017, OR = 13.757),* HLA-B* ^*∗*^ *2704*/^*∗*^ *5801* (*P* = 0.022, OR = 6.872),* HLA-B* ^*∗*^ *3501*/^*∗*^ *4403* (*P* = 0.021, OR = 30.269), and* HLA-B* ^*∗*^ *3905*/^*∗*^ *5801* (*P* = 0.032, OR = 24.697).

## 4. Discussion

Although the causes and mechanisms of ASD were unclear, the genetic and environment factors were believed to play an important role in the pathophysiology of the ASD. It was clear that no single genetic locus was the sole cause of this disorder. Recently, the genome-wide association studies revealed a number of rare mutations. Many of these genes encode proteins integral to formation, refinement, maintenance, function, and/or plasticity of synapses in the CNS; others encode proteins traditionally thought to play roles exclusively in the immune system, including MHC genes [18]. The interruption in MHC expression in the developing brain caused by mutations and/or immune deregulation may contribute to the altered brain connectivity and function characteristic of autism [4, 5, 19]. In addition, increased levels of cytokines in the plasma were found to be linked with greater impairments in characteristic ASD behavioral domains including social interaction and communication, as well as associated features such as aberrant behaviors [11].

Moreover, the* A2-B44* and* A2-B51* haplotypes were two times more frequent in autistic subjects [16]. In Egypt autistic children study found that autistic children had significant higher frequency of* HLA-DRB*1^*∗*^ *11* allele and lower frequency of* HLA-DRB*1^*∗*^ *03* allele than healthy-matched-children control (*P* < 0.001). Acquisition of* HLA-DRB*1^*∗*^ *11* and absence of* HLA-DRB*1^*∗*^ *03* had significant risk of association with autism and* HLA-DRB*1^*∗*^ *11* had a significant risk for association with a family history of autoimmunity in autistic children [20]. Moreover, Chien et al. 2012 suggested that* HLA-DRB1* gene might be associated with autism in Han Chinese and found that* DR4*,* DR11,* and* DR14* had a different effect on intelligence and neuropsychology tests among autistic children [21].

In our study, the findings obtained have shown that* HLA-B* ^*∗*^ *13:02*,* HLA-B* ^*∗*^ *38:02*,* HLA-B* ^*∗*^ *44:03,* and* HLA-B* ^*∗*^ *56:01* alleles were significantly associated with autism. This data was similar to the previous study by Torres et al. which reported a higher prevalence of* HLA-B* ^*∗*^ *44* and* HLA-B* ^*∗*^ *51* among ASD children than controls [13]. Contradictory, the previous finding of Al-Hakbany et al. reported a higher prevalence of* HLA-B* ^*∗*^ *07* and* HLA-B* ^*∗*^ *51* among ASD children than controls [15]. In addition, we found that the* HLA-B* ^*∗*^ *18:02* and* HLA-B* ^*∗*^ *46:12* alleles were significantly higher among controls than autistic subjects which demonstrated that these alleles might play a protective role toward disease development. We found that only* HLA-B* ^*∗*^ *44:03* alleles in autistic patients showed a significant difference in* HLA-B* genotype when compared with normal group. It might be the influence on gene-gene interaction that would result in autism development. The finding of small possibility of* HLA-B* genotype is a limitation of this study. As the result of* HLA-B* genotype, there are quite a small number of* HLA-B* genotypes in control. Either small sample size or family members should be included in further study for proving that those genotypes are associated in autistic patients.

With the different results in* HLA-B* publication, most of genetic studies unavoidably increase the attention to the ethnic variation. This finding might serve as genetic markers for susceptibility to ASD in Thailand and reveals a cover-up of genetic effect on autism. However, the interaction between* HLA* and different infectious agents or environmental allergen across geographical regions remains of interest for clarification ASD etiology.

## 5. Conclusions

Our studies suggest that* HLA-B* ^*∗*^ *13:02* (*P* = 0.019, OR = 2.229),* HLA-B* ^*∗*^ *38:02* (*P* = 0.049, OR = 1.628),* HLA-B* ^*∗*^ *44:03* (*P* = 0.016, OR = 1.645), and* HLA-B* ^*∗*^ *56:01* (*P* = 1.78 × 10^−4^ OR = 4.927) alleles were significantly increased in autistic subjects compared with normal subjects. These alleles may have a consequence on ASD development. However, the understanding of how* HLA-B* alleles have an impact on autism still needs more investigation.

## Figures and Tables

**Table 1:** Characteristics of autistic patients (*n* = 364) and normal subjects (*n* = 952).

Characteristics	Autistic patients Mean ± SD	Normal subjects Mean ± SD
Age (years)	9.17 ± 4.99	45.36 ± 26.15
Gender		
Male	314 (86.26%)	667 (70.06%)
Female	50 (13.74%)	285 (29.93%)

**Table 2:** *HLA-B* allele frequency in autistic children and control group.

*HLA-B* alleles	Number of individuals (%)		OR (95% CI)	*P* value^*∗*^
	ASD *n* = 364 Allele frequencies (%)	Control *n* = 952 Allele frequencies (%)		
*B* ^*∗*^ *07:02*	3 (0.41)	19 (1.00)	0.411 (0.121–1.391)	0.153
*B* ^*∗*^ *07:05*	19 (2.61)	30 (1.58)	1.674 (0.936–2.993)	0.082
*B* ^*∗*^ *07:09*	0 (0.00)	1 (0.05)	0.871 (0.035–21.406)	0.933
*B* ^*∗*^ *07:13*	0 (0.00)	3 (0.16)	0.373 (0.019–7.228)	0.514
*B* ^*∗*^ *07:14*	0 (0.00)	1 (0.05)	0.871 (0.035–21.406)	0.933
*B* ^*∗*^ *07:18*	0 (0.00)	1 (0.05)	0.871 (0.035–21.406)	0.933
*B* ^*∗*^ *08:01*	4 (0.55)	11 (0.58)	0.951 (0.302–2.996)	0.931
*B* ^*∗*^ *08:02*	0 (0.00)	1 (0.05)	0.871 (0.035–21.406)	0.933
*B* ^*∗*^ *08:03*	1 (0.14)	1 (0.05)	2.618 (0.164–41.906)	0.496
*B* ^*∗*^ *08:12*	0 (0.00)	1 (0.05)	0.871 (0.035–21.406)	0.933
*B* ^*∗*^ *08:33*	0 (0.00)	1 (0.05)	0.871 (0.035–21.406)	0.933
*B* ^*∗*^ *13:01*	40 (5.49)	146 (7.67)	0.700 (0.488–1.004)	0.527
***B*** ^*∗*^ ***13:02***	**16 (2.20)**	**19 (1.00)**	**2.229 (1.140–4.359)**	**0.019**
*B* ^*∗*^ *13:03*	0 (0.00)	1 (0.05)	0.871 (0.035–21.406)	0.933
*B* ^*∗*^ *13:09*	0 (0.00)	1 (0.05)	0.871 (0.035–21.406)	0.933
*B* ^*∗*^ *13:28*	0 (0.00)	1 (0.05)	0.871 (0.035–21.406)	0.933
*B* ^*∗*^ *13:39*	0 (0.00)	2 (0.11)	0.522 (0.025–10.893)	0.675
*B* ^*∗*^ *14:02*	2 (0.27)	0 (0.00)	13.107 (0.629–273.362)	0.097
*B* ^*∗*^ *14:13*	0 (0.00)	1 (0.05)	0.871 (0.035–21.406)	0.933
*B* ^*∗*^ *15:01*	12 (1.65)	23 (1.21)	1.371 (0.678–2.769)	0.379
*B* ^*∗*^ *15:02*	55 (7.55)	160 (8.40)	0.891 (0.648–1.226)	0.477
*B* ^*∗*^ *15:03*	1 (0.14)	0 (0.00)	7.854 (0.319–193.017)	0.207
*B* ^*∗*^ *15:04*	1 (0.14)	5 (0.26)	0.522 (0.061–4.479)	0.554
*B* ^*∗*^ *15:06*	0 (0.00)	1 (0.05)	0.871 (0.035–21.406)	0.933
*B* ^*∗*^ *15:07*	1 (0.14)	3 (0.16)	0.872 (0.091–8.393)	0.905
*B* ^*∗*^ *15:11*	0 (0.00)	7 (0.37)	0.174 (0.010–3.044)	0.231
*B* ^*∗*^ *15:12*	3 (0.41)	7 (0.37)	1.121 (0.289–4.348)	0.868
*B* ^*∗*^ *15:13*	2 (0.27)	13 (0.68)	0.401 (0.090–1.780)	0.229
*B* ^*∗*^ *15:17*	1 (0.14)	10 (0.53)	0.261 (0.034–2.039)	0.200
*B* ^*∗*^ *15:18*	3 (0.41)	4 (0.21)	1.965 (0.439–8.804)	0.377
*B* ^*∗*^ *15:20*	2 (0.27)	0 (0.00)	13.107 (0.629–273.362)	0.097
*B* ^*∗*^ *15:21*	4 (0.55)	6 (0.32)	1.748 (0.492–6.211)	0.388
*B* ^*∗*^ *15:22*	0 (0.00)	1 (0.05)	0.871 (0.035–21.406)	0.933
*B* ^*∗*^ *15:25*	11 (1.51)	27 (1.42)	1.067 (0.526–2.161)	0.858
*B* ^*∗*^ *15:27*	2 (0.27)	2 (0.11)	2.619 (0.368–18.634)	0.336
*B* ^*∗*^ *15:31*	0 (0.00)	1 (0.05)	0.871 (0.035–21.406)	0.933
*B* ^*∗*^ *15:32*	1 (0.14)	8 (0.42)	0.326 (0.041–2.601)	0.291
*B* ^*∗*^ *15:35*	3 (0.41)	22 (1.16)	0.354 (0.106–1.186)	*0.092*
*B* ^*∗*^ *15:88*	0 (0.00)	1 (0.05)	0.871 (0.035–21.406)	*0.933*
*B* ^*∗*^ *18:01*	24 (3.30)	58 (3.05)	1.085 (0.669–1.759)	*0.741*
***B*** ^*∗*^ ***18:02***	**7 (0.96)**	**48 (2.52)**	**0.375 (0.169–0.836)**	***0.016***
*B* ^*∗*^ *18:09*	0 (0.00)	1 (0.05)	0.871 (0.035–21.406)	*0.933*
*B* ^*∗*^ *18:18*	0 (0.00)	1 (0.05)	0.871 (0.035–21.406)	*0.933*
*B* ^*∗*^ *27:03*	1 (0.14)	5 (0.26)	0.522 (0.061–4.479)	*0.554*
*B* ^*∗*^ *27:04*	13 (1.79)	37 (1.94)	0.917 (0.485–1.736)	*0.791*
*B* ^*∗*^ *27:06*	12 (1.65)	15 (0.79)	2.111 (0.983–4.531)	*0.055*
*B* ^*∗*^ *27:07*	0 (0.00)	1 (0.05)	0.871 (0.035–21.406)	*0.933*
*B* ^*∗*^ *27:61*	0 (0.00)	1 (0.05)	0.871 (0.035–21.406)	*0.933*
*B* ^*∗*^ *27:86*	0 (0.00)	1 (0.05)	0.871 (0.035–21.406)	*0.933*
*B* ^*∗*^ *35:01*	14 (1.92)	36 (1.89)	1.017 (0.546–1.898)	*0.957*
*B* ^*∗*^ *35:02*	1 (0.14)	2 (0.11)	1.308 (0.118–14.449)	*0.826*
*B* ^*∗*^ *35:03*	6 (0.82)	21 (1.10)	0.745 (0.299–1.854)	*0.527*
*B* ^*∗*^ *35:05*	9 (1.24)	39 (2.05)	0.599 (0.288–1.242)	*0.168*
*B* ^*∗*^ *35:08*	1 (0.14)	0 (0.00)	7.854 (0.319–193.017)	*0.207*
*B* ^*∗*^ *35:11*	0 (0.00)	1 (0.05)	0.871 (0.035–21.406)	*0.933*
*B* ^*∗*^ *35:58*	0 (0.00)	1 (0.05)	0.871 (0.035–21.406)	*0.933*
*B* ^*∗*^ *36:68*	2 (0.27)	0 (0.00)	13.107 (0.629–273.362)	*0.097*
*B* ^*∗*^ *37:01*	4 (0.55)	9 (0.47)	1.163 (0.357–3.789)	*0.802*
*B* ^*∗*^ *38:01*	5 (0.69)	4 (0.21)	3.285 (0.879–12.267)	*0.077*
***B*** ^*∗*^ ***38:02***	**27 (3.71)**	**44 (2.31)**	**1.628 (1.001–2.649)**	***0.049***
*B* ^*∗*^ *38:13*	0 (0.00)	1 (0.05)	0.871 (0.035–21.406)	*0.933*
*B* ^*∗*^ *38:17*	0 (0.00)	1 (0.05)	0.871 (0.035–21.406)	*0.933*
*B* ^*∗*^ *38:20*	0 (0.00)	1 (0.05)	0.871 (0.035–21.406)	*0.933*
*B* ^*∗*^ *38:22*	0 (0.00)	1 (0.05)	0.871 (0.035–21.406)	*0.933*
*B* ^*∗*^ *38:23*	0 (0.00)	4 (0.21)	0.289 (0.016–5.391)	*0.406*
*B* ^*∗*^ *39:01*	7 (0.96)	11 (0.58)	1.412 (0.561–3.554)	*0.463*
*B* ^*∗*^ *39:03*	0 (0.00)	3 (0.16)	0.373 (0.019–7.228)	*0.514*
*B* ^*∗*^ *39:05*	1 (0.14)	2 (0.11)	1.308 (0.118–14.449)	*0.826*
*B* ^*∗*^ *39:09*	6 (0.82)	13 (0.68)	1.209 (0.458–3.192)	*0.702*
*B* ^*∗*^ *39:15*	1 (0.14)	13 (0.68)	0.200 (0.026–1.532)	*0.121*
*B* ^*∗*^ *39:24*	1 (0.14)	6 (0.32)	0.435 (0.052–3.621)	*0.441*
*B* ^*∗*^ *40:01*	72 (9.89)	155 (8.14)	1.239 (0.923–1.661)	*0.153*
*B* ^*∗*^ *40:02*	10 (1.37)	24 (1.26)	1.091 (0.519–2.293)	*0.818*
*B* ^*∗*^ *40:03*	0 (0.00)	1 (0.05)	0.871 (0.035–21.406)	*0.933*
*B* ^*∗*^ *40:04*	5 (0.69)	4 (0.21)	3.285 (0.879–12.267)	*0.077*
*B* ^*∗*^ *40:06*	3 (0.41)	11 (0.58)	0.712 (0.198–2.559)	*0.603*
*B* ^*∗*^ *40:09*	0 (0.00)	1 (0.05)	0.871 (0.035–21.406)	*0.933*
*B* ^*∗*^ *40:10*	0 (0.00)	6 (0.32)	0.200 (0.011–3.563)	*0.274*
*B* ^*∗*^ *40:23*	0 (0.00)	1 (0.05)	0.871 (0.035–21.406)	*0.933*
*B* ^*∗*^ *40:59*	0 (0.00)	2 (0.11)	0.522 (0.025–10.893)	*0.675*
*B* ^*∗*^ *41:01*	0 (0.00)	2 (0.11)	0.522 (0.025–10.893)	*0.675*
*B* ^*∗*^ *41:10*	0 (0.00)	1 (0.05)	0.871 (0.035–21.406)	*0.933*
*B* ^*∗*^ *44:01*	0 (0.00)	1 (0.05)	0.871 (0.035–21.406)	*0.933*
*B* ^*∗*^ *44:02*	6 (0.82)	11 (0.58)	1.430 (0.527–3.881)	*0.482*
***B*** ^*∗*^ ***44:03***	**40 (5.49)**	**65 (3.41)**	**1.645 (1.099–2.463)**	***0.016***
*B* ^*∗*^ *44:43*	0 (0.00)	1 (0.05)	0.871 (0.035–21.406)	*0.933*
*B* ^*∗*^ *44:54*	1 (0.14)	0 (0.00)	7.854 (0.319–193.017)	*0.207*
*B* ^*∗*^ *46:01*	86 (11.81)	209 (10.98)	1.086 (0.832–1.419)	*0.543*
***B*** ^*∗*^ ***46:12***	**2 (0.27)**	**35 (1.84)**	**0.147 (0.035–0.613)**	***0.008***
*B* ^*∗*^ *46:16*	1 (0.14)	0 (0.00)	7.854 (0.319–193.017)	*0.207*
*B* ^*∗*^ *48:01*	3 (0.41)	7 (0.37)	1.121 (0.289–4.348)	*0.868*
*B* ^*∗*^ *48:03*	1 (0.14)	5 (0.26)	0.522 (0.061–4.479)	*0.554*
*B* ^*∗*^ *48:21*	0 (0.00)	3 (0.16)	0.373 (0.019–7.228)	*0.514*
*B* ^*∗*^ *50:01*	1 (0.14)	2 (0.11)	1.308 (0.118–14.449)	*0.826*
*B* ^*∗*^ *51:01*	24 (3.30)	68 (3.57)	0.921 (0.573–1.478)	*0.732*
*B* ^*∗*^ *51:02*	6 (0.82)	20 (1.05)	0.783 (0.313–1.957)	*0.600*
*B* ^*∗*^ *51:04*	1 (0.14)	6 (0.32)	0.435 (0.052–3.621)	*0.441*
*B* ^*∗*^ *51:06*	0 (0.00)	3 (0.16)	0.373 (0.019–7.228)	*0.514*
*B* ^*∗*^ *51:07*	1 (0.14)	0 (0.00)	7.854 (0.319–193.017)	*0.207*
*B* ^*∗*^ *51:43*	0 (0.00)	1 (0.05)	0.871 (0.035–21.406)	*0.933*
*B* ^*∗*^ *51:45*	0 (0.00)	1 (0.05)	0.871 (0.035–21.406)	*0.933*
*B* ^*∗*^ *52:01*	10 (1.37)	48 (2.52)	0.538 (0.271–1.070)	*0.077*
*B* ^*∗*^ *52:07*	1 (0.14)	0 (0.00)	7.854 (0.319–193.017)	*0.207*
*B* ^*∗*^ *52:11*	1 (0.14)	1 (0.05)	2.618 (0.164–41.906)	*0.496*
*B* ^*∗*^ *52:25*	0 (0.00)	1 (0.05)	0.871 (0.035–21.406)	*0.933*
*B* ^*∗*^ *53:17*	0 (0.00)	3 (0.16)	0.373 (0.019–7.228)	*0.514*
*B* ^*∗*^ *54:01*	14 (1.92)	26 (1.37)	1.416 (0.735–2.728)	*0.298*
*B* ^*∗*^ *54:04*	0 (0.00)	2 (0.11)	0.522 (0.025–10.893)	*0.675*
*B* ^*∗*^ *54:14*	0 (0.00)	1 (0.05)	0.871 (0.035–21.406)	*0.933*
*B* ^*∗*^ *54:16*	1 (0.14)	0 (0.00)	7.854 (0.319–193.017)	*0.207*
*B* ^*∗*^ *55:01*	1 (0.14)	9 (0.47)	0.289 (0.037–2.290)	*0.240*
*B* ^*∗*^ *55:02*	8 (1.10)	26 (1.37)	0.803 (0.362–1.781)	0.588
*B* ^*∗*^ *55:04*	0 (0.00)	2 (0.11)	0.522 (0.025–10.893)	0.675
*B* ^*∗*^ *55:10*	0 (0.00)	1 (0.05)	0.871 (0.035–21.406)	0.933
*B* ^*∗*^ *55:13*	1 (0.14)	0 (0.00)	7.854 (0.319–193.017)	0.207
*B* ^*∗*^ *55:23*	0 (0.00)	1 (0.05)	0.871 (0.035–21.406)	0.933
*B* ^*∗*^ *55:32*	0 (0.00)	1 (0.05)	0.871 (0.035–21.406)	0.933
*B* ^*∗*^ *55:44*	0 (0.00)	1 (0.05)	0.871 (0.035–21.406)	0.933
***B*** ^*∗*^ ***56:01***	**13 (1.79)**	**7 (0.37)**	**4.927 (1.958–12.399)**	**1.78 × **10^−4^
*B* ^*∗*^ *56:02*	0 (0.00)	4 (0.21)	0.289 (0.016–5.391)	0.406
*B* ^*∗*^ *56:03*	0 (0.00)	2 (0.11)	0.522 (0.025–10.893)	0.675
*B* ^*∗*^ *56:04*	0 (0.00)	6 (0.32)	0.200 (0.011–3.563)	0.274
*B* ^*∗*^ *56:12*	0 (0.00)	1 (0.05)	0.871 (0.035–21.406)	0.933
*B* ^*∗*^ *56:16*	2 (0.27)	1 (0.05)	5.242 (0.474–57.905)	0.176
*B* ^*∗*^ *57:01*	16 (2.20)	24 (1.26)	1.760 (0.929–3.333)	0.083
*B* ^*∗*^ *57:21*	1 (0.14)	2 (0.11)	1.308 (0.118–14.449)	0.826
*B* ^*∗*^ *58:01*	65 (8.93)	163 (8.56)	1.047 (0.775–1.415)	0.764
*B* ^*∗*^ *58:34*	1 (0.14)	0 (0.00)	7.854 (0.319–193.017)	0.207
*B* ^*∗*^ *58:42*	0 (0.00)	1 (0.05)	0.871 (0.035–21.406)	0.933
*B* ^*∗*^ *67:01*	0 (0.00)	1 (0.05)	0.871 (0.035–21.406)	0.933
*B* ^*∗*^ *73:01*	1 (0.14)	0 (0.00)	7.854 (0.319–193.017)	0.207

^*∗*^Chi-square test.

OR: odds ratio; 95% CI: confidence interval. The bold font in table refers to significant association of alleles.

**Table 3:** *HLA-B* genotype frequency in autistic patients compared with control group.

Genotypes	ASD *n* = 364 (%)	Control *n* = 952 (%)	OR (95% CI)	*P* value^*∗∗*^
*B* ^*∗*^ *1801/* ^*∗*^ *4402*	5 (1.38)	1 (0.10)	13.757 (1.602–118.158)	0.017
*B* ^*∗*^ *2704/* ^*∗*^ *5801*	5 (1.38)	2 (0.20)	6.872 (1.327–35.577)	0.022
*B* ^*∗*^ *3501/* ^*∗*^ *4403*	5 (1.38)	0 (0.00)	30.269 (1.669–548.816)	0.021
*B* ^*∗*^ *3905/* ^*∗*^ *5801*	4 (1.10)	0 (0.00)	24.697 (1.326–459.879)	0.032

^*∗∗*^Chi-square test. OR: odds ratio; 95% CI: confidence interval.
